# Anaerobic derivates of mitochondria and peroxisomes in the free-living amoeba *Pelomyxa schiedti* revealed by single-cell genomics

**DOI:** 10.1186/s12915-022-01247-w

**Published:** 2022-03-01

**Authors:** Kristína Záhonová, Sebastian Cristian Treitli, Tien Le, Ingrid Škodová-Sveráková, Pavla Hanousková, Ivan Čepička, Jan Tachezy, Vladimír Hampl

**Affiliations:** 1 Department of Parasitology, Faculty of Science, Charles University, BIOCEV, Vestec, Czech Republic; 2 Institute of Parasitology, Biology Centre, Czech Academy of Sciences, České Budějovice (Budweis), Czech Republic; 3 Department of Biochemistry, Faculty of Natural Sciences, Comenius University, Bratislava, Slovakia; 4 Department of Zoology, Faculty of Science, Charles University, Prague, Czech Republic

**Keywords:** *Pelomyxa*, Mitochondrion-related organelle, Hydrogenosome, Anaerobic peroxisome, Anaerobiosis, FeS cluster assembly, Single-cell genomics

## Abstract

**Background:**

Mitochondria and peroxisomes are the two organelles that are most affected during adaptation to microoxic or anoxic environments. Mitochondria are known to transform into anaerobic mitochondria, hydrogenosomes, mitosomes, and various transition stages in between, collectively called mitochondrion-related organelles (MROs), which vary in enzymatic capacity. Anaerobic peroxisomes were identified only recently, and their putatively most conserved function seems to be the metabolism of inositol. The group Archamoebae includes anaerobes bearing both anaerobic peroxisomes and MROs, specifically hydrogenosomes in free-living *Mastigamoeba balamuthi* and mitosomes in the human pathogen *Entamoeba histolytica*, while the organelles within the third lineage represented by *Pelomyxa* remain uncharacterized.

**Results:**

We generated high-quality genome and transcriptome drafts from *Pelomyxa schiedti* using single-cell omics. These data provided clear evidence for anaerobic derivates of mitochondria and peroxisomes in this species, and corresponding vesicles were tentatively identified in electron micrographs. In silico reconstructed MRO metabolism harbors respiratory complex II, electron-transferring flavoprotein, a partial TCA cycle running presumably in the reductive direction, pyruvate:ferredoxin oxidoreductase, [FeFe]-hydrogenases, a glycine cleavage system, a sulfate activation pathway, and an expanded set of NIF enzymes for iron-sulfur cluster assembly. When expressed in the heterologous system of yeast, some of these candidates localized into mitochondria, supporting their involvement in the MRO metabolism. The putative functions of *P. schiedti* peroxisomes could be pyridoxal 5′-phosphate biosynthesis, amino acid and carbohydrate metabolism, and hydrolase activities. Unexpectedly, out of 67 predicted peroxisomal enzymes, only four were also reported in *M. balamuthi*, namely peroxisomal processing peptidase, nudix hydrolase, inositol 2-dehydrogenase, and d-lactate dehydrogenase. Localizations in yeast corroborated peroxisomal functions of the latter two.

**Conclusions:**

This study revealed the presence and partially annotated the function of anaerobic derivates of mitochondria and peroxisomes in *P. schiedti* using single-cell genomics, localizations in yeast heterologous systems, and transmission electron microscopy. The MRO metabolism resembles that of *M. balamuthi* and most likely reflects the state in the common ancestor of Archamoebae. The peroxisomal metabolism is strikingly richer in *P. schiedti*. The presence of *myo*-inositol 2-dehydrogenase in the predicted peroxisomal proteome corroborates the situation in other Archamoebae, but future experimental evidence is needed to verify additional functions of this organelle.

**Supplementary Information:**

The online version contains supplementary material available at 10.1186/s12915-022-01247-w.

## Background

Transition to life in low-oxygen environments requires significant modifications of cell biochemistry and organellar makeup. Several lineages of protists have undergone such transitions and exemplify partly convergent solutions [1–3]. Mitochondria and peroxisomes have been most significantly remodeled in this process, as they are the key places of oxygen-dependent metabolism and oxygen detoxification.

Mitochondria are double-membrane-bound organelles, which arose from the engulfment of members of a prokaryotic lineage related to alphaproteobacteria [2, 4–6]. Since then, they have diverged into a range of categories [1] and a plethora of transitional forms [7, 8], collectively designated as mitochondrion-related organelles (MROs), while only a single case of complete loss has been reported [9]. A substantial number of typical mitochondrial functionalities, such as oxidative phosphorylation; carbon, amino acid, and fatty acid metabolism; iron-sulfur (FeS) cluster assembly; homeostasis; and apoptosis, have been reduced to various extents in different MROs [10–12].

Peroxisomes are bound by a single membrane and characterized by a highly conserved set of proteins (peroxins) essential for their biogenesis [13, 14]. Their matrix content and consequently the repertoire of metabolic pathways is highly variable, reflecting a high degree of versatility in peroxisomal functions [15]. Most frequently, they possess oxidases reducing molecular oxygen to hydrogen peroxide (H_2_O_2_), and catalase for H_2_O_2_ detoxification. Not surprisingly, they are absent from most anaerobes, such as *Giardia* and *Trichomona*s [16]; however, anaerobic peroxisomes were recently reported from two Archamoebae, namely *Mastigamoeba balamuthi* and *Entamoeba histolytica* [17, 18].

Archamoebae represents a clade of microaerophilic protists nested within a broader group of predominantly aerobic amoebozoans [19, 20] represented, e.g., by *Dictyostelium discoideum* (Eumycetozoa), known to bear a classical aerobic mitochondrion [21], or by their more distant amoebozoan relative *Acanthamoeba castellanii* (Centramoebida) with mitochondria potentially adapted to periods of anaerobiosis and exhibiting a highly complex proteome [12, 22]. Small to almost inconspicuous MROs have been characterized in two Archamoebae, the parasitic *E. histolytica* and the free-living *M. balamuthi*. The only known function of the *E. histolytica* mitosome is production and export of activated sulfate—phosphoadenosine-5′-phosphosulfate (PAPS) [23]. The metabolic capacity of the *M. balamuthi* hydrogenosome is substantially broader, involving pyruvate and amino acid metabolism, ATP production, and FeS cluster assembly [24–26]. Another adaptation of both amoebae to the low oxygen environment is represented by anaerobic peroxisomes that lack catalase and enzymes of β-oxidation, and which are predicted to contain a diverse set of enzymes with only eight being common to both species, including *myo*-inositol 2-dehydrogenase, long-chain fatty acid-CoA ligase, and malate dehydrogenase [17, 18].

*Pelomyxa* is a free-living archamoeba that is distantly related to both *M. balamuthi* and *E. histolytica* [19], and so it represents a valuable point for tracing the evolution of anaerobic adaptations. *Pelomyxa schiedti* was previously isolated from freshwater sediments of lake Skadar in Albania [27]. It is characterized by a peripheral ring of chromatin granules in its nucleus and the presence of numerous prokaryotic endosymbionts in its cytosol [27]. There is a single report on MROs in the giant species *P. palustris* [28], but their metabolism is unknown. Using methods of single-cell -omics and electron microscopy, we bring clear evidence for the presence of both MROs and peroxisomes in its smaller cousin *P. schiedti* [27].

## Results and discussion

### General features of assemblies

**Fig. 1:**
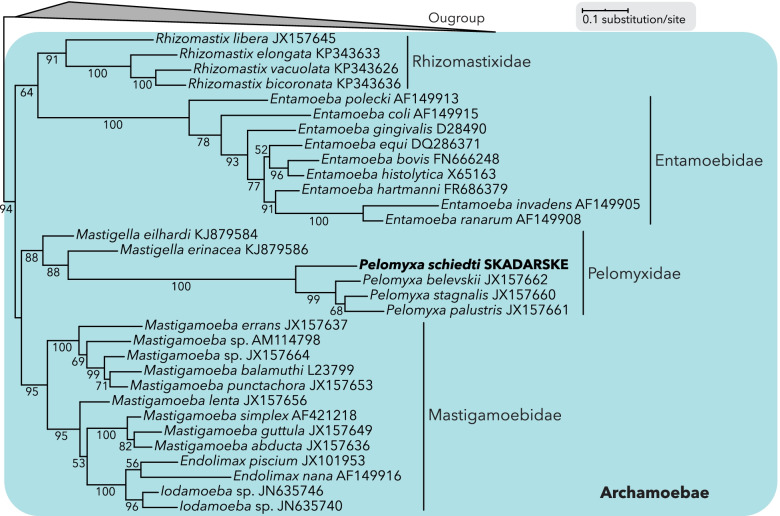
Phylogenetic analysis of amoebozoan 18S rDNA. Maximum-likelihood tree places *Pelomyxa schiedti* in monophyletic Pelomyxidae group inside monophyletic Archamoebae. Standard bootstrap support values are shown when ≥ 50%. Outgroup was collapsed for simplicity (for the full tree, see Additional file 2: Fig. S1)

*Pelomyxa schiedti* genome assembly generated from seven micromanipulated cells had a total length of 52.4 Mb and contained 5337 scaffolds with an N50 = 51,552 bp and 19,876 predicted proteins (Additional file 1: Table S1). We identified a single small subunit ribosomal RNA gene (18S rDNA). In our 18S phylogeny, *P. schiedti* was sister to other *Pelomyxa* species inside the Pelomyxidae clade (88% standard bootstrap support) within a robust clade (94% standard bootstrap) of Archamoebae (Fig. 1; Additional file 2: Fig. S1). The decontaminated transcriptome assembly of 76.6 Mb comprised 43,993 contigs. BUSCO was used to estimate the completeness of assemblies and to compare them to the *M. balamuthi* genome (Additional file 2: Fig. S2; Additional file 1: Table S1). The transcriptome contained 83.2% complete and 2.0% fragmented BUSCO genes, while in the genome-derived proteome, the proportions were 81.9% and 3.6%, respectively. With 82.8% complete and 3.0% fragmented genes [29], the completeness of *M. balamuthi* data was comparable. Duplicated BUSCOs represented 36.0% and 8.6% in the transcriptome and genome assembly, respectively, reflecting a higher number of contigs, or the presence of isoforms, in the former. It should be noted that for non-model eukaryotes, which *Pelomyxa* certainly is, BUSCO completeness is not expected to reach 100%, because some orthologues might be absent and/or diverged beyond recognition. Altogether, our analyses showed considerably high completeness of both assemblies.

*P. schiedti* genes encompassed 149,016 introns (Additional file 1: Table S1), which accounts for a density of 7.43 introns/gene, almost twice as high as *M. balamuthi* (3.74). While protists’ genomes have usually lower intron densities, several organisms in IntronDB [30] exhibit similar intron density as *Pelomyxa*, e.g., the choanoflagellates *Monosiga brevicolis* (6.53) and *Salpingoeca rosetta* (7.44), the chromerid *Vitrella brassicaformis* (7.45), and the chlorarachniophyte *Bigelowiella natans* (7.85). The vast majority of introns (98.41%) contained canonical GT-AG boundaries, 1.59% possessed GC-AG boundaries, and one had an unusual GT-GG intron boundary (Additional file 1: Table S1). Similar frequencies of intron boundaries are observed in *M. balamuthi* (Additional file 1: Table S1 and [29]).

### Putative MRO proteome

**Fig. 2:**
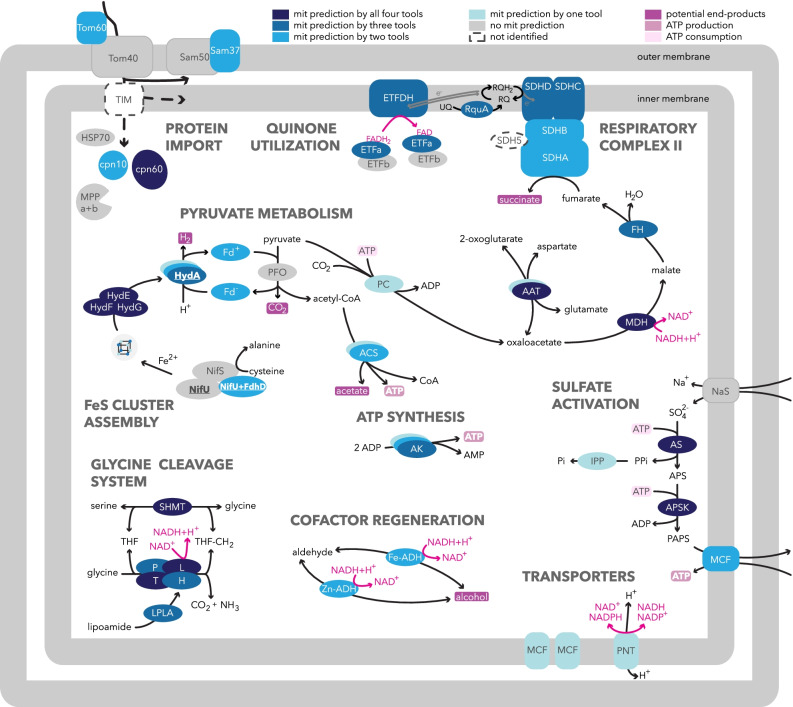
Overview of the *Pelomyxa schiedti* MRO metabolism. Proteins were identified by BLAST or HMMER searches, and their intracellular localization was predicted by TargetP, PSORT II, MultiLoc2, and NommPred tools. Confidence of MRO localization is enhanced by shades of blue as explained in graphical legend above the scheme. Multiple copies of a protein are shown as overlapping ovals. Proteins that localized in *S. cerevisiae* mitochondria (Fig. 3) are in bold and underlined. AAT, aspartate aminotransferase; ACO, aconitase; ACS, acetyl-CoA synthetase; AK, adenylate kinase; APS, adenosine-5′-phosphosulfate; APSK, adenosine-5′-phosphosulfate kinase; AS, ATP sulfurylase; cpn10, chaperonin 10; cpn60, chaperonin 60; CoA, coenzyme A; ETFa, electron transferring flavoprotein subunit alpha; ETFb, electron transferring flavoprotein subunit beta; ETFDH, electron transferring flavoprotein dehydrogenase; Fe-ADH, iron-containing alcohol dehydrogenase; Fd, ferredoxin; FdhD, formate dehydrogenase accessory sulfurtransferase; FH, fumarase; H, glycine cleavage system H protein; HSP70, heat shock protein 70; HydA, [FeFe]-hydrogenase; HydE, hydrogenase maturase; HydF, hydrogenase maturase; HydG, hydrogenase maturase; IPP, inorganic pyrophosphatase; L, glycine cleavage system L protein; LPLA, lipoamide protein ligase; MCF, mitochondrial carrier family; MDH, malate dehydrogenase; MPP a+b, mitochondrial processing peptidase subunit alpha and beta; NaS, sodium/sulfate symporter; NifS, cysteine desulfurase; NifU, scaffold protein; P, glycine cleavage system P protein; PAPS, 3′-phosphoadenosine 5′-phosphosulfate; PC, pyruvate carboxylase; PFO, pyruvate:ferredoxin oxidoreductase; PNT, pyridine nucleotide transhydrogenase; RQ, rodoquinone; RQH2, rhodoquinol; RquA, RQ methyltransferase; SAM, sorting and assembly machinery; SDH5, succinate dehydrogenase assembly factor; SDH5, succinate dehydrogenase assembly factor; SDHA, succinate dehydrogenase subunit A; SDHB, succinate dehydrogenase subunit B; SDHC, succinate dehydrogenase subunit C; SDHD, succinate dehydrogenase subunit D; SHMT, serine hydroxymethyltransferase; T, glycine cleavage system T protein; THF, tetrahydrofolate; THF-CH2, N^5^,N^10^-methylenetetrahydrofolate; TOM/TIM, translocase of outer/inner membrane; UQ, ubiquinone; Zn-ADH, zinc-containing alcohol dehydrogenase

The major focus of this study was to test for the presence of, and then to characterize, the putative proteomes of the MRO and peroxisome of *P. schiedti.* We used a combined approach to search for proteins possibly involved in MRO metabolism and biogenesis by (i) retrieving homologues of MRO- or mitochondrion-targeted proteins of *E. histolytica*, *M. balamuthi*, and *A. castelanii*; (ii) predicting N-terminal mitochondrial targeting sequences (NTS) by four tools to be able to identify potentially novel pathways; and (iii) localizing selected candidate proteins in the heterologous system of *Saccharomyces cerevisiae*. The resulting predicted MRO proteome based on three lines of evidence consisted of 46 proteins (Fig. 2; Additional file 1: Table S2) and putatively provides the functionalities described below.

### Protein import machinery

Despite sensitive HMMER searching, we identified only four subunits of the outer membrane translocase (TOM) and the sorting and assembly machinery (SAM) complexes (Fig. 2). Tom40 (Additional file 1: Table S2) forms a pore through which proteins are translocated [31]. Tom60, a shuttle receptor of matrix and membrane proteins, was thought to be *Entamoeba*-specific [32], but its presence in *P. schiedti* (Additional file 1: Table S2) suggests that it may be a universal component of the TOM complex in all archamoebal species. The SAM complex is required for protein insertion into the outer membrane after its translocation via the TOM complex [31]. We found Sam50, which forms a central channel of the complex, and Sam37, which participates in protein insertion (Additional File 1: Table S2). All four proteins had corresponding domains predicted by InterProScan. However, many homologues of the canonical opisthokont subunits were missing (Additional file 1: Table S2), as were all parts of the translocase system of the inner membrane (TIM), and so the mechanism of protein import across this membrane remains unknown. This situation resembles that of other Archamoebae [24, 29, 33], suggesting that archamoebal translocons are in general either highly streamlined and/or contain highly divergent or lineage-specific subunits as reported from trichomonads or trypanosomes [34, 35].

**Fig. 3:**
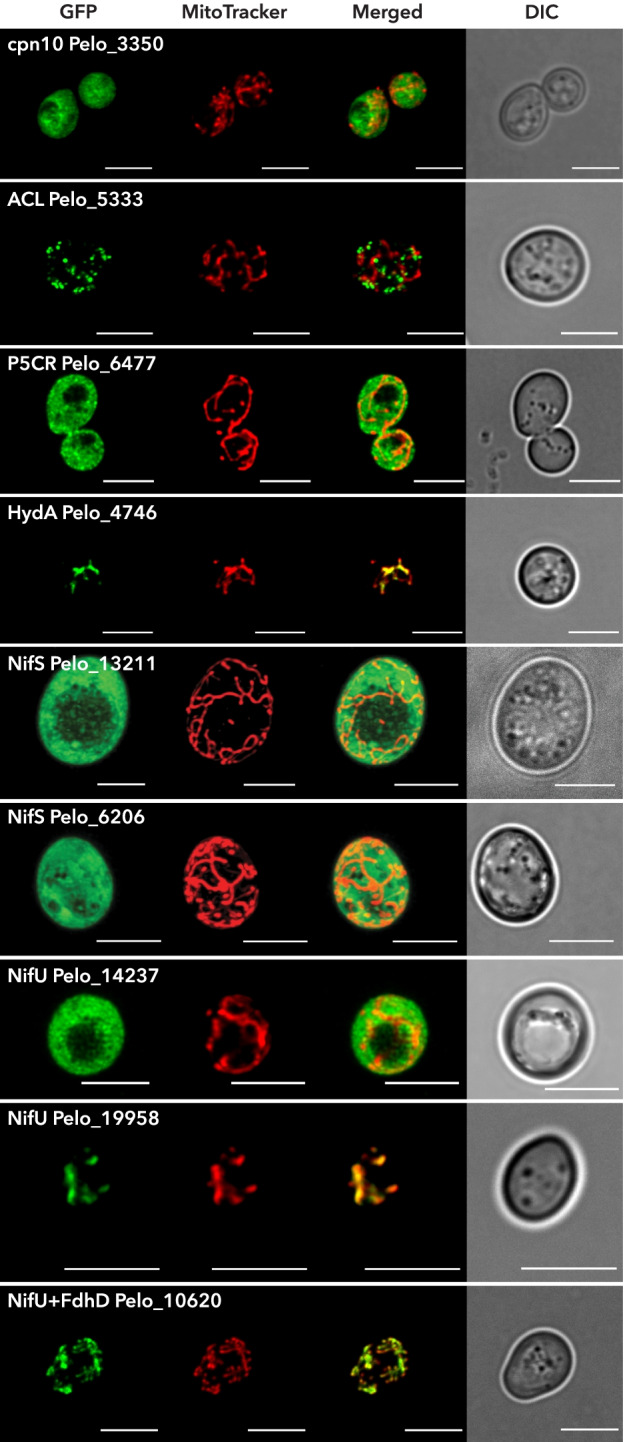
Heterologous localization of candidate MRO proteins of *Pelomyxa schiedti*. C-terminally GFP-tagged proteins were expressed in *Saccharomyces cerevisiae*. Mitochondria were stained with MitoTracker. Note that gene sequences were amplified from cDNA using specific primers (Additional file 1: Table S4), except for P5CR that was synthesized in vitro. ACL, ATP-citrate lyase; cpn10, chaperonin 10; DIC, differential interference contrast; FdhD, formate dehydrogenase accessory sulfurtransferase; HydA, [FeFe]-hydrogenase; NifS, cysteine desulfurase; NifU, scaffold protein; P5CR, pyrroline-5-carboxylate reductase. Scale bar, 5 μm

Enzymes involved in proteolytic processing (matrix processing peptidase) and folding (chaperonins cpn10 and cpn60) were present in our predicted proteome. Although cpn10 is a strictly specific mitochondrial protein, which is undoubtedly localized in the MRO, heterologous expression in *S. cerevisiae* failed to localize this protein into its mitochondria (Fig. 3). This fact may be explained by the loss of positive charge in the NTS of some hydrogenosome and mitosome targeted proteins, which is crucial for protein import into yeast mitochondria [36]. At the same time, it suggests that observations from heterologous systems, particularly the absence of targeting, should be interpreted with caution.

HSP70 was detected in 14 copies; none of them confidently predicted to be mitochondrial (Additional file 1: Table S2). Phylogenetic analysis revealed a single putative mtHSP70 sequence (Pelo_10550) branching sister to *M. balamuthi* mtHSP70 within the mitochondrial clade (Additional file 2: Fig. S3). The other HSP70 paralogues fell into the ER or cytosolic clades, the latter being diversified in ten copies.

Although we have probably revealed only a fragment of the inventory needed for the protein import into the *P. schiedtii* MRO, the presence of mitochondrial hallmarks—Tom40, Sam50, mtHSP70, cpn60, and cpn10—conclusively shows that the MRO is truly present.

### Tricarboxylic acid cycle and electron transport chain

*Pelomyxa schiedti* encodes four enzymes of the tricarboxylic acid (TCA) cycle possessing NTS (Additional file 1: Table S2) and catalyzing consecutive reactions. Instead of citrate synthase, the common TCA enzyme, we identified an ATP citrate lyase (ACL) homologous to human ACL (EC: 2.3.3.8), which harbors citryl-CoA synthetase alpha and citryl-CoA lyase domains, and thus may provide oxaloacetate for the reductive direction of the TCA. However, ACL was only weakly predicted to localize in the MRO (i.e., by a single tool) and, when heterologously expressed in yeast, ACL localized into vesicles that did not overlap with stained mitochondria (Fig. 3). Thus, ACL was excluded from the list of putative MRO proteins. Other identified proteins, fumarate hydratase (fumarase/FH), malate dehydrogenase (MDH), and four subunits of the succinate dehydrogenase complex (SDH/complex II/CII), were robustly predicted to localize in the MRO. These proteins are common for both oxidative and reductive TCA, and the absence of the CII subunit SDH5/SDHAF involved in the flavination of the SDHA subunit [37] is likely common for Archamoebae as it is absent also from *M. balamuthi* [25].

Homologues of *A. castellanii* respiratory complexes were not identified, except for the aforementioned CII and a quinone-dependent electron-transferring flavoprotein (ETF; Additional file 1: Table S2). Both soluble subunits, alpha (ETFa) and beta (ETFb), and the membrane-bound ETF dehydrogenase (ETFDH), are present but only ETFDH and ETFa contain a recognizable NTS. It has been proposed in *M. balamuthi* that electrons may be transferred in an unknown direction between ETF and rhodoquinone (RQ), a quinone molecule with a lower electron potential than ubiquinone [3, 38]. RQ is synthesized in *M. balamuthi* by a hydrogenosomal methyltransferase dubbed RquA [38], which was also detected in *P. schiedti* (Additional file 1: Table S2). RQ presence allows the delivery of electrons to CII that could function as fumarate reductase [39] producing succinate, the putative end product of the partial reversed TCA in both archamoebal species [25], which may be secreted as in *Trypanosoma* [40].

### Pyruvate and ATP metabolism

Pyruvate is oxidatively decarboxylated in aerobic mitochondria to acetyl-coenzyme A (CoA) by the pyruvate dehydrogenase (PDH) complex. In most MROs, PDH is substituted by pyruvate:ferredoxin oxidoreductase (PFO), pyruvate:NADP^+^ oxidoreductase (PNO), or pyruvate formate lyase (PFL) [2]. We identified six copies of PFO and one copy of PNO in the *P. schiedti* genome, all without a predicted NTS (Additional file 1: Table S2). However, one of the *P. schiedti* PFOs was sister to one of the putatively hydrogenosomal PFOs in *M. balamuthi* [25] (Additional file 2: Fig. S4) and hence might represent a PFO homologue that operates in the *P. schiedti* MRO. Another pyruvate-metabolizing enzyme predicted to function in the MRO is pyruvate carboxylase (PC; Additional file 1: Table S2), which catalyzes carboxylation of pyruvate to oxaloacetate [41], a substrate of MDH. In *M. balamuthi*, pyruvate may be produced by the activity of NAD^+^-dependent d-lactate dehydrogenases (D-LDH), one of which localizes in the hydrogenosome and the other in the peroxisome [17, 25]. However, *P. schiedti* bears only one homologue of D-LDH that was predicted to localize in its peroxisomes (Additional file 1: Table S3). Since we have no evidence for any pyruvate-producing enzyme in MRO, we hypothesize that pyruvate is most likely imported from the cytosol.

Two ATP-synthesizing enzymes are putatively present. Acetyl-CoA synthetase (ACS), an enzyme converting acetyl-CoA to acetate, CoA, and ATP, was found in eight copies, four of which possessed a putative NTS. ATP may also be formed by an adenylate kinase (AK) catalyzing the interconversion of adenine nucleotides. Three of the six AKs that we identified are putatively localized in the MRO (Additional file 1: Table S2). In this respect, the situation resembles that of the *M. balamuthi* hydrogenosome, in which both enzymes produce ATP [25]. A third putative source of ATP is the antiport against PAPS produced via the sulfate activation pathway (see below).

### Amino acid metabolism

The glycine cleavage system (GCS) is at least partially retained in many MROs [42]. It consists of four enzymes (H-, L-, T-, and P-protein) and methylates tetrahydrofolate (THF) while decomposing glycine into CO_2_ and ammonia. THF methylation is also provided by serine hydroxymethyltransferase (SHMT) [43]. We identified all GCS enzymes and SHMT in *P. schiedti*, all with predicted NTS (Fig. 2; Additional file 1: Table S2). L-protein was present in two copies with only one bearing an NTS, similarly to *M. balamuthi*, in which the function of the cytosolic copy is unknown [25]. Lipoamide protein ligase (LPLA), necessary for lipoamide attachment to GCSH, was present with an NTS. The resulting N^5^,N^10^-methylenetetrahydrofolate (CH_2_-THF) is an intermediate in one-carbon metabolism and a cofactor for the synthesis of pyrimidines and methionine in both mitochondria and the cytosol. Two cytosolic enzymes requiring this cofactor, B12-dependent methionine synthase, and THF dehydrogenase/cyclohydrolase, were detected (Additional file 1: Table S2). Glycine can be produced in mitochondria from threonine by threonine dehydrogenase (TDH) and alpha-amino-beta-ketobutyrate CoA ligase (AKL) [44] but both proteins lack a recognizable NTS in *P*. *schiedti* (Additional file 1: Table S2). Consistently, TDH activity was measured only in the cytosolic fraction of *M. balamuthi* [25]. It is highly probable that this pathway operates in the cytosol of *P. schiedti* and that glycine is imported into the MRO.

To our surprise, pyrroline-5-carboxylate reductase (P5CR) was predicted to be mitochondrion-targeted by one predictor (Additional file 1: Table S2). This enzyme could potentially be involved in the proline degradation pathway, which operates in the mitochondria of the distantly related amoebozoan *A. castellanii* [22]. As P5CR represents the only part of the pathway with localization signal whatsoever (Additional file 1: Table S2), we rather tested its localization in the *S. cerevisiae* heterologous system. There, the enzyme was clearly localized in the cytosol (Fig. 3), diminishing the probability of localizing the proline degradation pathway in the *P. schiedti* MRO. Moreover, further examination of the P5CR sequence revealed the presence of a peroxisomal targeting signal (Additional file 1: Table S3), which was masked in localization experiments by a C-terminal tag, and thus precluded its peroxisomal targeting in yeast. For these reasons, we included P5CR among putative peroxisomal proteins.

### Cofactor regeneration

NADH produced by GCS would be reoxidized in most mitochondria by NADH dehydrogenases in the electron transport chain [45]. Since the electron transport chain is absent in *P. schiedti*, we explored other pathways for cofactor regeneration. One possibility is fermentation of aldehydes to alcohols by alcohol dehydrogenases [46] putatively targeted to the MRO (Additional file 1: Table S2). Another option is the reductive partial TCA cycle, running from oxaloacetate to succinate, consuming not only NADH but also electrons from ETFDH via CII [47]. Oxaloacetate may be produced from pyruvate by PC with ATP consumption or by the action of aspartate aminotransferase (Additional file 1: Table S2); however, the source of 2-oxoglutarate and aspartate and the fate of glutamate is unclear due to the absence of a glutamate-aspartate antiporter (Additional file 1: Table S2).

Pyridine nucleotide transhydrogenase (PNT) is predicted to localize to the MRO by a single predictor (Additional file 1: Table S2). PNT usually localizes in the inner mitochondrial membrane and pumps protons while transferring electrons between NADH and NADPH [48]. PNT is present in *M. balamuthi* and *E. histolytica* [24, 49]; however, in *E. histolytica*, it was shown to localize outside mitosomes [50], which calls into question its MRO localization in other Archamoebae.

The *P. schiedti* MRO contains an ETF complex and a set of [FeFe]-hydrogenases, two additional electron sinks. ETF and ETFDH proteins are known to use electrons from the oxidation of fatty acids, a pathway that is absent in the *P. schiedti* MRO. [FeFe]-hydrogenases uptake electrons from reduced ferredoxins and produce molecular hydrogen. Three of the six detected hydrogenases bear putative NTS, and indeed, the one with the strongest prediction (Pelo_4746) was targeted to *S. cerevisiae* mitochondria (Fig. 3). Hydrogenases contain catalytic H clusters, and their maturation is dependent on maturases HydE, HydF, and HydG [51], which are all present and contain NTS (Additional file 1: Table S2). Meanwhile, reduced ferredoxin could originate from pyruvate oxidation.

### Iron-sulfur cluster assembly

Mitochondria usually house the iron-sulfur (FeS) cluster assembly (ISC) pathway inherited from the alphaproteobacterial ancestor and serve for the maturation of both mitochondrial and cytosolic FeS proteins [52]. Some organisms, including Archamoebae, have replaced it with another pathway via horizontal gene transfer [26, 53]. *M. balamuthi* bears two copies of the nitrogen fixation (NIF) system, both comprising NifS and NifU proteins. While one pair of NIFs operates in the cytosol, the other localizes in the hydrogenosome [26]. In *E. histolytica*, only the cytosolic copy has been retained [25].

**Fig. 4:**
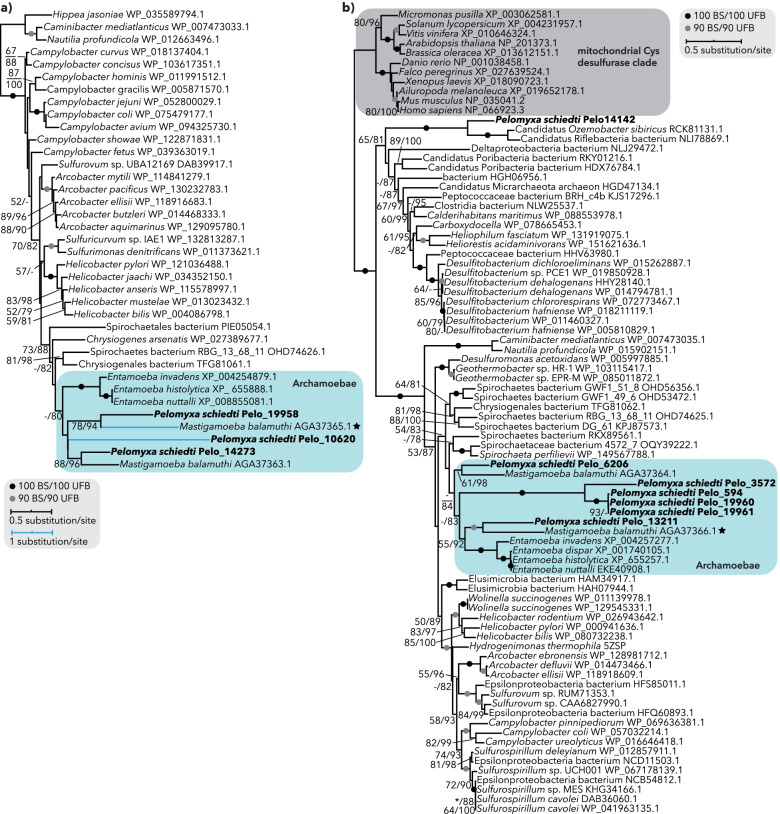
Analyses of NIF system components. **a**, **b** Maximum-likelihood phylogenetic trees show that *Pelomyxa schiedti* possesses orthologues of hydrogenosomal and cytosolic NifU (**a**) and NifS (**b**) proteins from *Mastigamoeba balamuthi*. Hydrogenosomal proteins of *M. balamuthi* are marked with stars. Tree support was estimated with standard (BS) and ultrafast bootstrapping (UFB). The tree topology shown is from the ultrafast bootstrap analysis. Support values for < 50% BS and < 75% UFB are denoted by a dash (-), whereas asterisk (*) marks the topology that does not exist in the respective analysis. Fully supported nodes are shown as black circles, nodes with ≥ 90% BS and ≥ 90% UFB are shown as grey circles, while nodes that were not supported are without any value

In the *P. schiedti* MRO, hydrogenases and their maturases HydE and HydF, SDH, ferredoxin, and PFO are putative clients for the NIF system and the situation with the gene inventory of the NIF system is by far the most complex among the investigated Archamoebae. We identified seven NifS and three NifU proteins, of which only one NifU (Pelo_10620) contained predicted NTS (Additional file 1: Table S2). Interestingly, this protein consists of a NifU N-terminal domain fused to a formate dehydrogenase accessory sulfurtransferase (FdhD) C-terminal domain (Additional file 2: Fig. S5a). In *Escherichia coli*, FdhD transfers sulfur from IscS to formate dehydrogenase (FdhF) and is thus essential for the FdhF activity [54]. *P. schiedti* indeed encodes a FdhF homologue but without an NTS (Additional file 1: Table S2). In the NifU phylogeny (Fig. 4a), the NifU domain of the fusion protein formed a long branch within a moderately supported (80% ultrafast bootstrap) clade of all archamoebal NifUs. The other *P. schiedti* NifU sequences branched sister to hydrogenosomal and cytosolic *M. balamuthi* homologues. All three *P. schiedti* NifU sequences contained conserved cysteine residues (Additional file 2: Fig. S5b) necessary for their function [55].

None of the seven NifS proteins was predicted to localize in the MRO (Additional file 1: Table S2). Two sequences were identical but incomplete at their C-termini and could not be completed by read mapping or PCR amplification. In our phylogenetic analysis (Fig. 4b), Pelo_13211 and Pelo_6206 branched sister to the hydrogenosomal and cytosolic *M. balamuthi* sequences, respectively. Pelo_14142 was sister to Candidati Riflebacteria species, and the remaining four sequences formed a long branch nested within the Archamoebae clade. All amino acid residues required for functioning [56] were present in both of the *P. schiedti* sequences that were sister to *M. balamuthi* (Additional file 2: Fig. S5c).

This phylogenetic pattern offers an elegant hypothesis in which NifS Pelo_6206 and NifU Pelo_14273 act in the cytosol, while NifS Pelo_13211 and NifU Pelo_19958 act in the MRO. The remaining NifS copies might be functional partners of the NifU-FdhD fusion protein (Pelo_10620). Our experiments with heterologous localization in *S. cerevisiae* indeed revealed mitochondrial localization of the NifU Pelo_19958, but surprisingly also of the NifU-FdhD fusion protein (Pelo_10620), and at the same time, both hypothesized NifS sequences were localized in the cytosol (Fig. 3). Although the situation with the NIF system in *P. schiedti* cells remains unclear, the mitochondrial localization of two NifU homologues in yeast supports the presence of the FeS cluster assembly in the *P. schiedti* MRO with the identity of its NifS partner unresolved.

### Sulfate activation pathway

The sulfate activation pathway produces PAPS, which is necessary for sulfolipid synthesis [23]. It is present in *E. histolytica* [23, 57] and *M. balamuthi* [25] MROs, and we also identified all of its components in *P. schiedti* (Fig. 2; Additional file 1: Table S2). This pathway requires two transporters. A sodium/sulfate symporter (NaS) is necessary for substrate delivery; however, its homologues in *P. schiedti* (Additional file 1: Table S2) are unrelated to *E. histolytica* mitosomal NaS [23] (Additional file 2: Fig. S6a) making their role unclear. The PAPS exporter belongs to the mitochondrial carrier family (MCF) and, indeed, one of the *P. schiedti* MCF proteins branched sister to a clade of PAPS transporters of *E. histolytica* and *M. balamuthi* [29, 58] (Additional file 2: Fig. S6b). As this transporter exchanges PAPS with ATP, it plays a role in supplementing the ATP pool in the MRO, yet it cannot provide a net ATP gain, because two ATP molecules are required for the production of one PAPS.

### Anaerobic peroxisomes

**Fig. 5:**
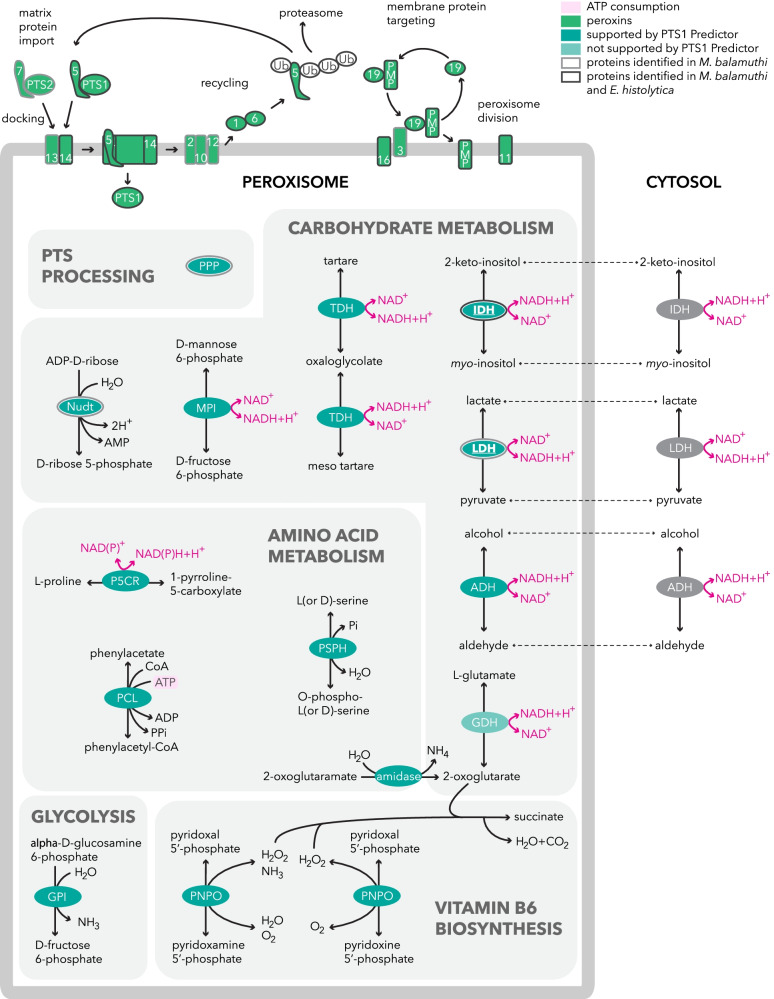
Overview of the *Pelomyxa schiedti* peroxisomal metabolism. Peroxins were identified by BLAST searches. Peroxisomal matrix proteins were predicted by searching for peroxisomal targeting signals (PTS; Additional file 1: Table S3). Proteins that localized in *S. cerevisiae* peroxisomes (Fig. 6) are in bold and underlined. AAT, aspartate aminotransferase; ADH, alcohol dehydrogenase; GDH, glutamate dehydrogenase; GPI, glucose-6-phosphate isomerase; IDH, *myo*-inositol 2-dehydrogenase; LDH, d-lactate dehydrogenase; MPI, mannose-6-phosphate isomerase; Nudt, nudix hydrolase; P5CR, pyrroline-5-carboxylate reductase; PCL, phenylacetate-CoA ligase; PMP, peroxisomal membrane protein; PNPO, pyridoxamine 5′-phosphate oxidase; PPP, peroxisomal processing peptidase; PTS, peroxisomal targeting signal; PSPH, phosphoserine phosphatase; TDH, tartrate dehydrogenase. Numbers indicate peroxins

We also investigated the presence of anaerobic peroxisomes, which were recently characterized in *M. balamuthi* and *E. histolytica* [17, 18]*. P. schiedti* encodes genes for 13 proteins required for peroxisome biogenesis (peroxins, Pexs) strongly supporting the presence of peroxisomes. The identified Pexs include Pex5 and Pex7, which are required for the recognition of peroxisomal targeting signals 1 and 2 (PTS1 and PTS2), respectively; Pex13 and 14, which mediate protein import; Pex1, 2, 6, 10, and 12, which are receptor-recycling Pexs; Pex3, 16, and 19, which are involved in protein import to the peroxisomal membrane; and Pex11, which participates in peroxisome fission (Fig. 5; Additional file 1: Table S3). Interestingly, the sets of Pexs in *P. schiedti* and *M. balamuthi* are identical, with the exception of two paralogs of Pex11 being found in *M. balamuthi* [17]. Thus, it seems that this set of Pexs is common to free-living Archamoebae. In contrast, in the parasitic *Entamoeba*, either the set of Pexs was reduced to only seven components (*E. histoytica*), or peroxisomes were completely lost (*E. invadens*) [18].

**Fig. 6:**
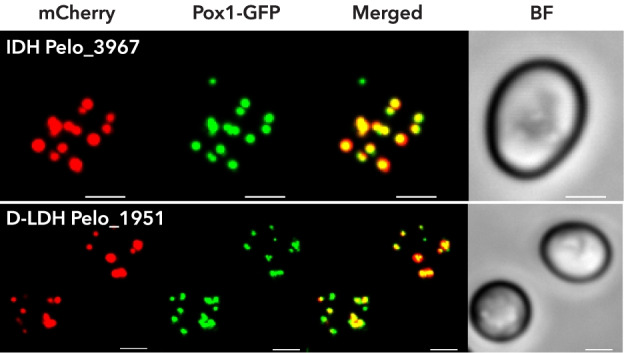
Heterologous localization of candidate peroxisomal proteins of *Pelomyxa schiedti*. Proteins were expressed in *Saccharomyces cerevisiae* with mCherry-tag at their N-terminus. GFP-tagged Pox1 was used as peroxisomal marker. DIC, differential interference contrast; D-LDH, d-lactate dehydrogenase; IDH, *myo*-inositol 2-dehydrogenase; Pox1, protein acyl-CoA oxidase. Scale bar, 2 μm

Prediction of putative peroxisomal matrix proteins based on the presence of PTS1/PTS2, revealed 67 candidates (Fig. 5; Additional file 1: Table S3). Of these, only four candidates had previously been found in the anaerobic peroxisomes of *M. balamuthi*. These include peroxisomal processing peptidase (PPP), *myo*-inositol 2-dehydrogenase (*myo*-IDH), nudix hydrolase, and d-lactate dehydrogenase (D-LDH), all of which had clear support for localization in *P. schiedti* peroxisomes (Additional file 1: Table S3). Two of them (*myo*-IDH and D-LDH) were selected for heterologous expression with an N-terminal mCherry tag in yeast, expressing the integrated GFP-tagged peroxisomal marker protein acyl-CoA oxidase (Pox1). Fluorescence microscopy revealed that both *myo*-IDH and D-LDH colocalized with Pox1 in round organelles in support of our predictions (Fig. 6).

Unlike in *M. balamuthi*, *P. schiedti* peroxisomes possibly contain pyridoxamine 5′-phosphate oxidase (PNPO; Additional file 1: Table S3), which utilizes molecular oxygen as an electron acceptor to catalyze the last step of the pyridoxal 5′-phosphate (PLP) biosynthesis with concomitant formation of ammonia and H_2_O_2_. The presence of PNPO raises the question of how H_2_O_2_ is detoxified as typical antioxidant enzymes, such as catalase and peroxidase, are not present. However, H_2_O_2_ could also be decomposed nonenzymatically by antioxidants, such as 2-oxoglutarate, in which the ketone group of the α-carbon atom reacts with H_2_O_2_ to form succinate, CO_2_, and water [59]. Indeed, *P. schiedti* contains a putative glutamate dehydrogenase that may produce 2-oxoglutarate and that possesses an –SKL triplet, a typical PTS1 (Additional file 1: Table S3) although its peroxisomal targeting was not supported by the PTS predictor. The other proteins with predicted peroxisomal localization include several enzymes for amino acid synthesis and degradation, carbohydrate metabolism, and hydrolases, albeit without clear biochemical context. More experimental studies are required to verify our predicted localizations and to delineate the function of peroxisomes in *P. schiedti*.

### Electron microscopy

**Fig. 7:**
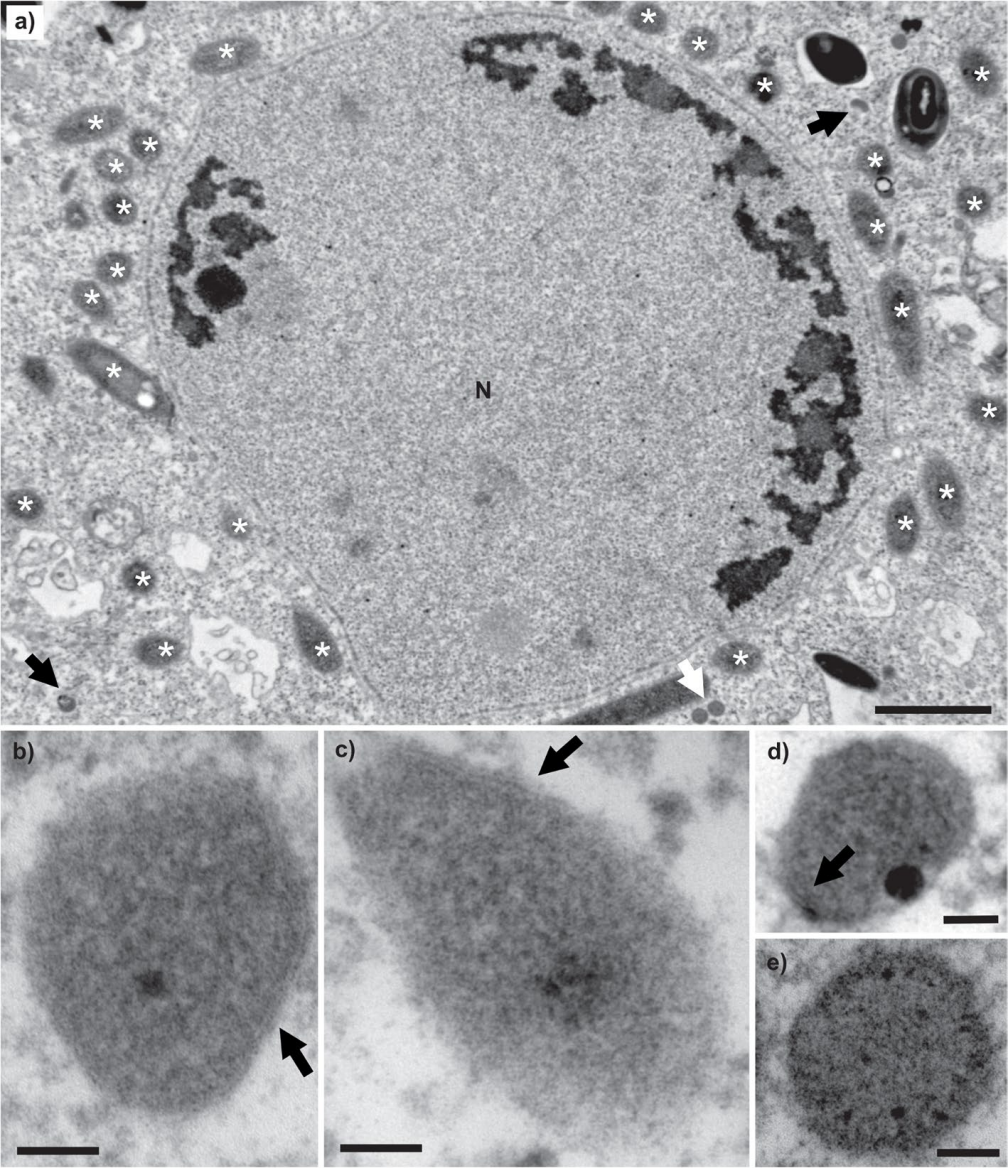
Transmission electron micrograph of *Pelomyxa schiedti*, ultra-thin sections. **a** Nuclear area. N, nucleus; black arrow, putative mitochondrion-related organelle; white arrow, small dense body (putative peroxisome); asterisk (*), prokaryotic endosymbiont. **b**–**d** High magnification of putative mitochondrion-related organelle. Specimens were fixed with 0.1 M cacodylate (**b**, **c**) or PHEM buffer (**d**). Black arrow, bounding double membrane. **e** High magnification of small dense body (putative peroxisome). Scale bars, 400 nm for **a** and 50 nm for **b**–**e**

Finally, we were interested in whether the two organelles described by the genomic data can be visualized by transmission electron microscopy. Careful inspection of micrographs revealed two populations of small vesicles, one bounded by a double membrane and the other by a single membrane (Fig. 7). We ascribe them respectively to putative MROs and peroxisomes characterized in this work in silico but leave confirmation of this for further studies.

## Conclusions

Our bioinformatic survey of the putative proteome of the *Pelomyxa schiedti* MRO revealed several interesting insights and opened many questions for further investigation of this amoeba, which represent another, understudied lineage of Archamoebae. Most importantly, *P. schiedti* clearly does harbor an MRO with a very streamlined or lineage-specific set of protein translocases, and peroxisomes with a set of 13 soluble and membrane-associated peroxins. The MRO metabolism predicted in silico is similar to that of *M. balamuthi*, and it probably resembles the situation in the common free-living ancestor of this group of amoebae. Still, the situation with FeS cluster assembly in this amoeba seems rather complex and interesting as it contains the most diverse set of NIF pathway proteins of all previously investigated Archamoebae. These proteins very likely provide parallel FeS synthesis in MRO and cytosol, but in addition to this and uniquely in eukaryotes, some of them may be involved in the activation of formate dehydrogenase as seen in some prokaryotes. *P. schiedti* anaerobic peroxisomes, similarly to those of *M. balamuthi* and *E. histolytica*, lack enzymes for the β-oxidation of fatty acids and catalase, but the predicted set of enzymes differs considerably from other Archamoebae. Only four enzymes are shared between at least two species, and two of these (*myo*-IDH and D-LDH) were shown to localize in yeast peroxisomes, making them credible peroxisomal enzymes not only in *P. schiedti* but also in other Archamoebae. Although the role of these peroxisomes needs to be clarified, they seem to be metabolically diversified among Archamoebae, and very distinct from their aerobic counterparts.

## Material and methods

### Cell culture

Polyxenic (and polyeukaryotic) cultures of *Pelomyxa schiedti* strain SKADARSKE were maintained in Sonneborn’s *Paramecium* medium [60] as described previously [27].

### Genome and transcriptome sequencing and assembly

Genome sequencing was performed from whole genome amplified DNA (WGA). Individual cells were picked by micromanipulation and washed twice in Trager U media [61]. Genomic DNA was amplified using the Illustra Single Cell GenomiPhi DNA Amplification Kit (GE Healthcare Life Sciences) according to the manufacturer’s protocol and purified using ethanol precipitation. To confirm the presence of eukaryotic DNA, a fragment of the actin gene was amplified by PCR using specific primers (Additional file 1: Table S4). Sequencing libraries from seven positive samples were prepared using the Illumina TruSeq DNA PCR-Free kit (Illumina). Samples Pelo2 and Pelo5 were sequenced using the Illumina MiSeq (2 × 300 bp; Genomic Core Facility, Faculty of Science) and Nanopore (Oxford Nanopore Technologies) systems; samples P1–P5 were sequenced using the Illumina HiSeq X (Macrogen, Inc.). The Nanopore library was prepared using the Oxford Nanopore Technologies ligation sequencing kit (SQK-LSK108) from 4 μg of T7 endonuclease I-treated (New England Biolabs) WGA. Sequencing was performed using a R9.4.1 Spot-On Flow cell (FLO-MIN106) for 48 h.

For transcriptome sequencing, single cells of *P. schiedti* were washed twice in Trager U medium, and transcriptomes were generated using the Smart Seq2 [62] protocol with 19 cycles of amplification. Five libraries were prepared using the Nextera XT DNA Library Preparation Kit (Illumina) and sequenced on the Illumina MiSeq platform (PE 2 × 300 bp; Genomic Core Facility, Faculty of Science).

Raw Illumina DNA- and RNA-Seq reads were quality- and adapter-trimmed using BBDuk v36.92 (part of BBTools suite: https://jgi.doe.gov/data-and-tools/bbtools/). Firstly, individual single-cell genome assemblies for Pelo2, Pelo5, and P1–P5 were generated with SPAdes v3.11.1 [63] using single-cell (--sc) mode and a *k*-mer size of 127. As all 18S rDNAs extracted from individual assemblies were identical, all reads (i.e., Illumina HiSeqs and MiSeq, and Nanopore) were assembled together by SPAdes v3.11.1 using --sc and *k*-mers of 21, 33, 55, 77, 99, and 121. The resulting assembly was binned and decontaminated using tetraESOM [64] and a BLASTing strategy as described previously [65]. The final assembly was scaffolded using the P_RNA_scaffolder [66]. Prediction was done using Augustus v3.3.1 [67], and further improved by PASA and EVM [68] using our transcriptomic data. RNA-Seq reads were assembled using Trinity v2.6.5 [69] with default parameters, and contaminants were removed by BLASTing against the decontaminated genome assembly. RNA-Seq reads were mapped to the transcriptome using Bowtie2 v2.3.0 [70] and to the genome using HISAT2 v2.0.5 [71]. Genome and transcriptome completeness were assessed using BUSCO v3 with the eukaryota_odb9 dataset [72].

### Sequence searches and localization predictions

Proteins predicted to localize in the *M. balamuthi* hydrogenosome, *E. histolytica* mitosome, and *A. castellanii* mitochondria served as queries for BLAST v2.6.0+ [73] searches in *P. schiedti* assemblies. Sensitive searches for components of the TOM/TIM machinery were done using HMMER v3.3 [74]. Protein domains were predicted by InterProScan [75] as implemented in Geneious Prime v2020.2.3 [76].

Potentially mitochondrion-targeted proteins were identified using TargetP v2 [77], PSORT II [78], MultiLoc2 [79], and NommPred [80] tools. Since *P. schiedti* does not harbor plastid, the plant setting from TargetP and MultiLoc2 was omitted. NommPred was used in the MRO and in the *Dictyostelium* settings. A protein was considered as mitochondrial if predicted by at least one setting of MultiLoc2 or NommPred.

Peroxins were identified by BLAST searches using *M. balamuthi* queries. Peroxisomal matrix proteins were predicted by searching for peroxisomal targeting signals (PTS). The tripeptides SRI and [SAP][KR][LM] (excluding AKM, PKM, and PRM) were used to search for the C-terminal PTS1. A proline at position -3 and a methionine at position -1 were included based on experimental verification in *M. balamuthi* [17]. Two nanopeptides R[LI](x5)HL were used for N-terminal PTS2 searches [81]. All putative transmembrane proteins determined by TMHMM Server v2.0 [82], were filtered out. PTS1 candidates were submitted to the PTS1 Predictor [83] using the GENERAL function evaluating twelve C-terminal residues.

### Phylogenetic analyses

The 18S rRNA gene dataset was aligned by MAFFT v7 [84] server with the G-INS-i algorithm using default settings. The alignment was manually edited in BioEdit v7.0.4.1 [85] resolving 1437 positions. A phylogenetic tree was constructed using maximum-likelihood by RAxML v8.0.0 [86] under the GTRGAMMAI model, 100 starting trees, and 1000 standard bootstrap pseudoreplicates.

For selected proteins, datasets were aligned by MAFFT v7.313 [84], trimmed by trimAl v1.4 [87], and maximum-likelihood trees were inferred by IQ-TREE v1.6.8 [88] using the posterior mean site frequency method [89] and the LG+C20+F+G model, with the guide tree inferred under the LG+F+G model. Branch supports were obtained by the ultrafast bootstrap approximation [90] with 1000 replicates. For NIF components, standard bootstrapping with 100 replicates was used as well.

### Localization assays

Most of the localized genes were amplified from cDNA using specific primers (Additional file 1: Table S4) and PrimeSTAR® Max DNA Polymerase (Takara Bio, Inc.) premix. Only the P5CR (Pelo_6477) gene sequence was synthesized in vitro (GenScript). For mitochondrial localization, the genes were cloned into the pUG35 vector containing C-terminal green fluorescence protein (GFP) and transformed into *S. cerevisiae* strain BY4742 using the lithium acetate method [91]. Transformants were grown on a selective medium without uracil (SD-URA) at 30 °C. For localization, the transformed cells were incubated with MitoTracker Red CMXRos (1:10,000; Thermo Fisher Scientific) for 10 min, followed by two washes with PBS, and mounted in 1% low-melting agarose. Cells were imaged using a Leica SP8 confocal microscope. Deconvolution was performed using Huygens Professional v17.10 and ImageJ v1.50b. For heterologous expression of peroxisomal candidates in yeast, the genes were cloned into the pTVU100 vector for protein expression, with an N-terminal mCherry tag, in *S. cerevisiae* strain BY4742:POX1-EGFP, expressing the GFP-tagged peroxisomal marker protein acyl-CoA oxidase (Pox1) [17, 92]. Selected transformants were inoculated in oleate medium to stimulate peroxisome formation, followed by microscopy [17].

### Transmission electron microscopy

A grown culture of *P. schiedti* was pelleted by centrifugation and fixed for 1 h on ice with 2.5% glutaraldehyde in 0.1 M cacodylate buffer (pH 7.2). After washing in 0.1 M cacodylate buffer, the cells were postfixed for 1 h on ice with 1% OsO_4_. After washing with distilled water, the fixed cells were dehydrated in a graded series of ethanol, transferred to acetone, and embedded in EPON resin. Alternatively, the protocol was modified several times using different buffers (PHEM and PBS buffer) and a fixation time of 30 min. Ultrathin sections were prepared on an ultramicrotome (Reichert-Jung Ultracut E) with a diamond knife. Sections were stained with uranyl acetate and lead citrate and examined using a JEOL 1011 transmission electron microscope.

## Supplementary Information

**Additional file 1:** **Table S1.** Statistics of *Pelomyxa schiedti* assemblies were compared with those of *Mastigamoeba balamuthi*. **Table S2.** Proteins targeted to MRO of *Pelomyxa schiedti*. Localization of proteins was predicted by several tools, as listed in columns E - J. Mitochondrial predictions are highlighted by white text on blue background. Column K shows final inferred prediction of localization. Abbreviations: cyt, cytosolic; ER, endoplasmic reticulum; extracell, extracellular; mit, mitochondrial; nuc, nuclear; Other, other localization; perox, peroxisomal; PM, plasma membrane; sec, secretory system; SP, signal peptide; -, protein not localized in MRO; +, protein localized in MRO; +?, protein localized in MRO with low confidence. **Table S3.** Proteins required for peroxisome biogenesis and targeted to peroxisome. Proteins identified in *Mastigamoeba balamuthi* are highlighted by white text on blue background. Proteins were considered peroxisome-targeted, if they contained PTS1 (SRI or [SAP][KR][LM]) or PTS2 motif (R[LIV](x5)HL), and/or were predicted by PTS1 predictor [83]. **Table S4.** Primers used in this study.

**Additional file 2:** **Figure S1.** Phylogenetic analysis of amoebozoan 18S rDNA. Maximum-likelihood tree places *Pelomyxa schiedti* in monophyletic Pelomyxidae group inside monophyletic Archamoebae. Standard bootstrap support values shown when ≥ 50%. **Figure S2.** BUSCO analysis of *Pelomyxa schiedti* transcriptome and predicted proteins. Completeness of *P. schiedti* datasets were assessed using odb9_eukaryota dataset and compared with completeness of predicted proteins from *Mastigamoeba balamuthi*. **Figure S3.** Phylogenetic analysis of HSP70 proteins. Maximum-likelihood phylogenetic tree documents that one *Pelomyxa schiedti* HSP70 sequence is related to mitochondrial orthologues from other eukaryotes. Ultrafast bootstrap support values shown when ≥ 75%. ER, endoplasmic reticulum. **Figure S4.** Phylogenetic analysis of PFO enzymes. Maximum-likelihood phylogenetic tree identifies PFO version putatively operating in *Pelomyxa schiedti* MRO. Hydrogenosomal PFO copies of *Mastigamoeba balamuthi* marked with stars. Number in parentheses shows number of species in the collapsed clade. Ultrafast bootstrap support values shown when ≥ 75%. **Figure S5.** Sequences of *Pelomyxa schiedti* components of NIF system. a) *P. schiedti* protein Pelo_10620, composed of NifU N-terminal domain fused to FdhD (formate dehydrogenase accessory sulfurtransferase) C-terminal domain, as determined by InterProScan. b-c) Sequence alignment of NifU (b) and NifS (c) proteins from *P. schiedti* and *Mastigamoeba balamuthi* in comparison with bacterial homologues from *Thermotoga maritima*. Amino acid residues necessary for function of NifU and NifS are labeled according to graphical legend. **Figure S6.** Phylogenetic analysis of transporters involved in sulfate activation pathway. a) Phylogenetic analysis did not resolve which of the sodium/sulfate symporters of *Pelomyxa schiedti* is related to *Entamoeba histolytica* mitosomal transporter. b) Maximum-likelihood phylogenetic tree confirms one *P. schiedti* transporter as PAPS (3’-phosphoadenosine 5’-phosphosulfate) transporter, while two others belong to broader family of mitochondrial carrier transporters. Experimentally proven mitosomal transporters of *E. histolytica* marked with stars. Ultrafast bootstrap support values shown when ≥ 75%.

## Data Availability

All data needed to evaluate the conclusions in the paper are present in the paper and/or the supplementary information files. The raw sequencing data and final assemblies are available at NCBI (https://www.ncbi.nlm.nih.gov/) as BioProject PRJNA672820 [93].
