# Supplementary material for: Anaerobic derivates of mitochondria and peroxisomes in the free-living amoeba Pelomyxa schiedti revealed by single-cell genomics
Source: BMC Biol. 2022 Mar 1;20:56. doi: 10.1186/s12915-022-01247-w (PMC8887013; doi:10.1186/s12915-022-01247-w)
Supplement: Supplementary file 2 — Additional file 2: Figure S1. Phylogenetic analysis of amoebozoan 18S rDNA. Maximum-likelihood tree places Pelomyxa schiedti in monophyletic Pelomyxidae group inside monophyletic Archamoebae. Standard bootstrap support values shown when ≥ 50%. Figure S2. BUSCO analysis of Pelomyxa schiedti transcriptome and predicted proteins. Completeness of P. schiedti datasets were assessed using odb9_eukaryota dataset and compared with completeness of predicted proteins from Mastigamoeba balamuthi. Figure S3. Phylogenetic analysis of HSP70 proteins. Maximum-likelihood phylogenetic tree documents that one Pelomyxa schiedti HSP70 sequence is related to mitochondrial orthologues from other eukaryotes. Ultrafast bootstrap support values shown when ≥ 75%. ER, endoplasmic reticulum. Figure S4. Phylogenetic analysis of PFO enzymes. Maximum-likelihood phylogenetic tree identifies PFO version putatively operating in Pelomyxa schiedti MRO. Hydrogenosomal PFO copies of Mastigamoeba balamuthi marked with stars. Number in parentheses shows number of species in the collapsed clade. Ultrafast bootstrap support values shown when ≥ 75%. Figure S5. Sequences of Pelomyxa schiedti components of NIF system. a) P. schiedti protein Pelo_10620, composed of NifU N-terminal domain fused to FdhD (formate dehydrogenase accessory sulfurtransferase) C-terminal domain, as determined by InterProScan. b-c) Sequence alignment of NifU (b) and NifS (c) proteins from P. schiedti and Mastigamoeba balamuthi in comparison with bacterial homologues from Thermotoga maritima. Amino acid residues necessary for function of NifU and NifS are labeled according to graphical legend. Figure S6. Phylogenetic analysis of transporters involved in sulfate activation pathway. a) Phylogenetic analysis did not resolve which of the sodium/sulfate symporters of Pelomyxa schiedti is related to Entamoeba histolytica mitosomal transporter. b) Maximum-likelihood phylogenetic tree confirms one P. schiedti transporter as PAPS (3’-phosphoadeno [file 12915_2022_1247_MOESM2_ESM.pdf]

Anaerobic derivates of mitochondria and peroxisomes in the free-living amoeba *Pelomyxa schiedti* revealed by single-cell genomics

Kristína Záhonová[1,2], Sebastian Cristian Treitli[1], Tien Le[1], Ingrid Škodová-Sveráková[2,3], Pavla Hanousková[4], Ivan Čepička[4], Jan Tachezy[1], Vladimír Hampl[1]

[1] Department of Parasitology, Faculty of Science, Charles University, BIOCEV, Vestec, Czech Republic

[2] Institute of Parasitology, Biology Centre, Czech Academy of Sciences, České Budějovice (Budweis), Czech Republic

[3] Department of Biochemistry, Faculty of Natural Sciences, Comenius University, Bratislava, Slovakia

[4] Department of Zoology, Faculty of Science, Charles University, Prague, Czech Republic

Corresponding authors: kika.zahonova@gmail.com (KZ); vlada@natur.cuni.cz (VH)

Additional file 2: Figures S1 – S6

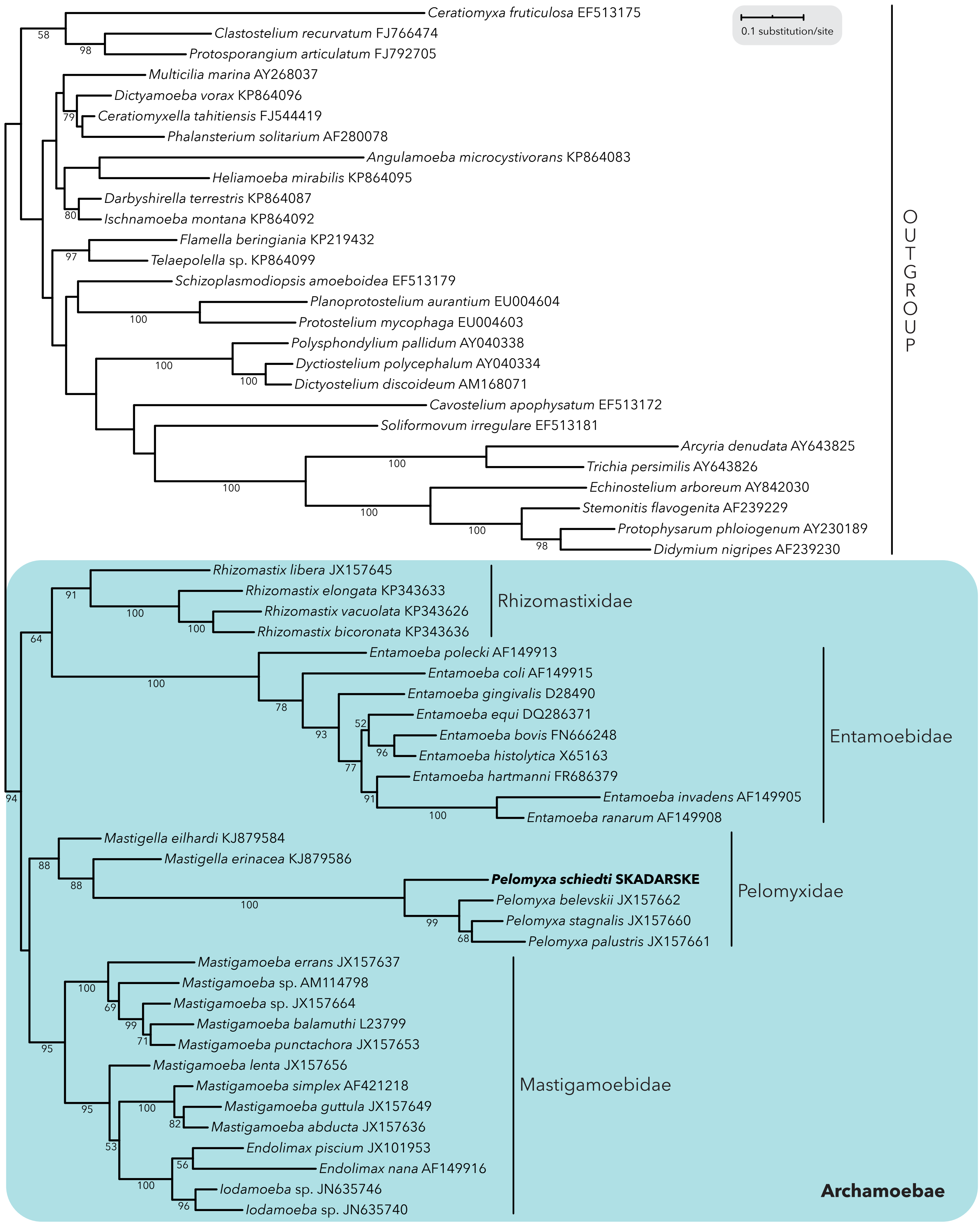

Fig. S1. Phylogenetic analysis of amoebozoan 18S rDNA. Maximum-likelihood tree places *Pelomyxa schiedti* in monophyletic Pelomyxidae group inside monophyletic Archamoebae. Standard bootstrap support values shown when ≥50%.

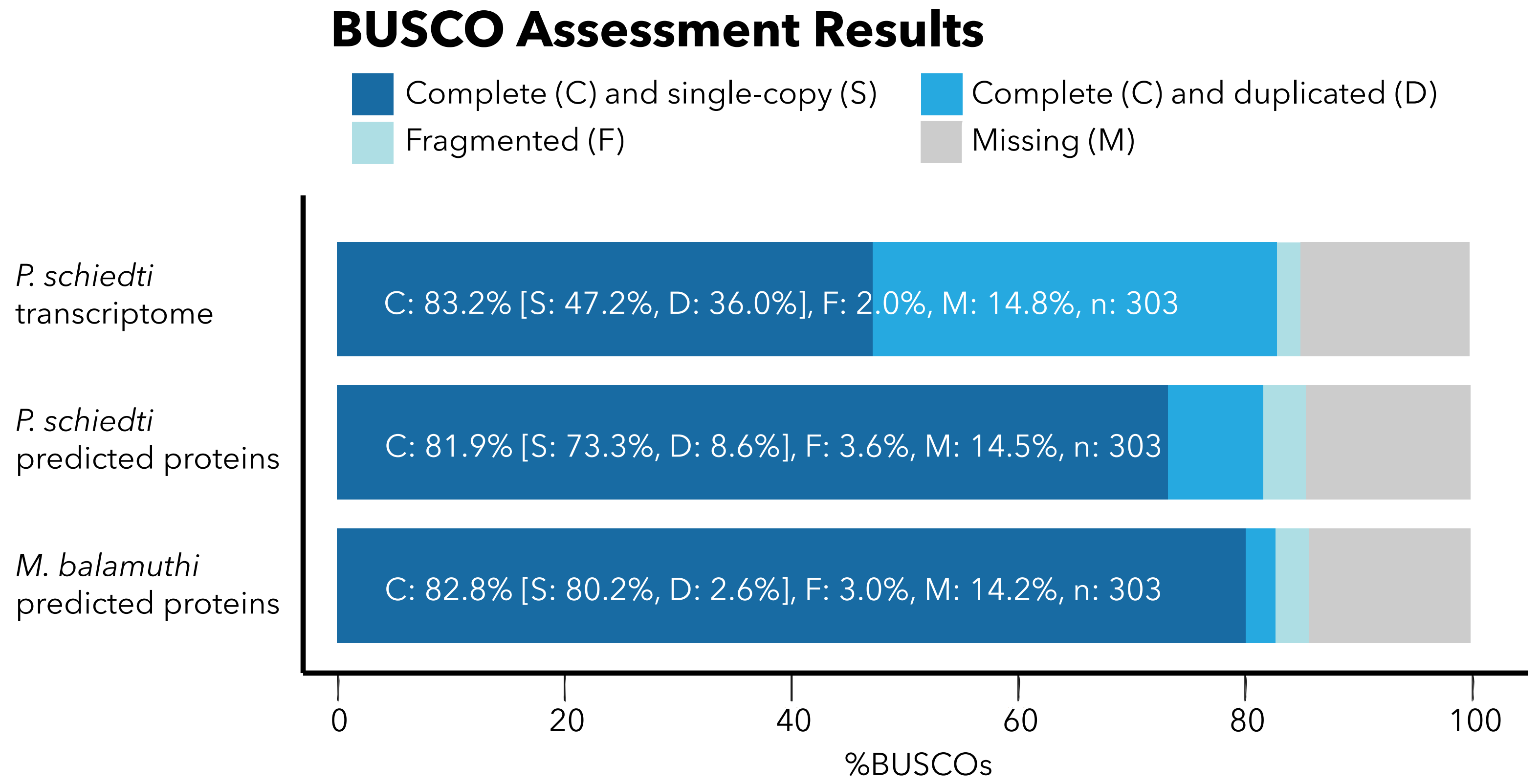

Fig. S2. BUSCO analysis of *Pelomyxa schiedti* transcriptome and predicted proteins. Completeness of *P. schiedti* datasets were assessed using odb9_eukaryota dataset and compared with completeness of predicted proteins from *Mastigamoeba balamuthi*.

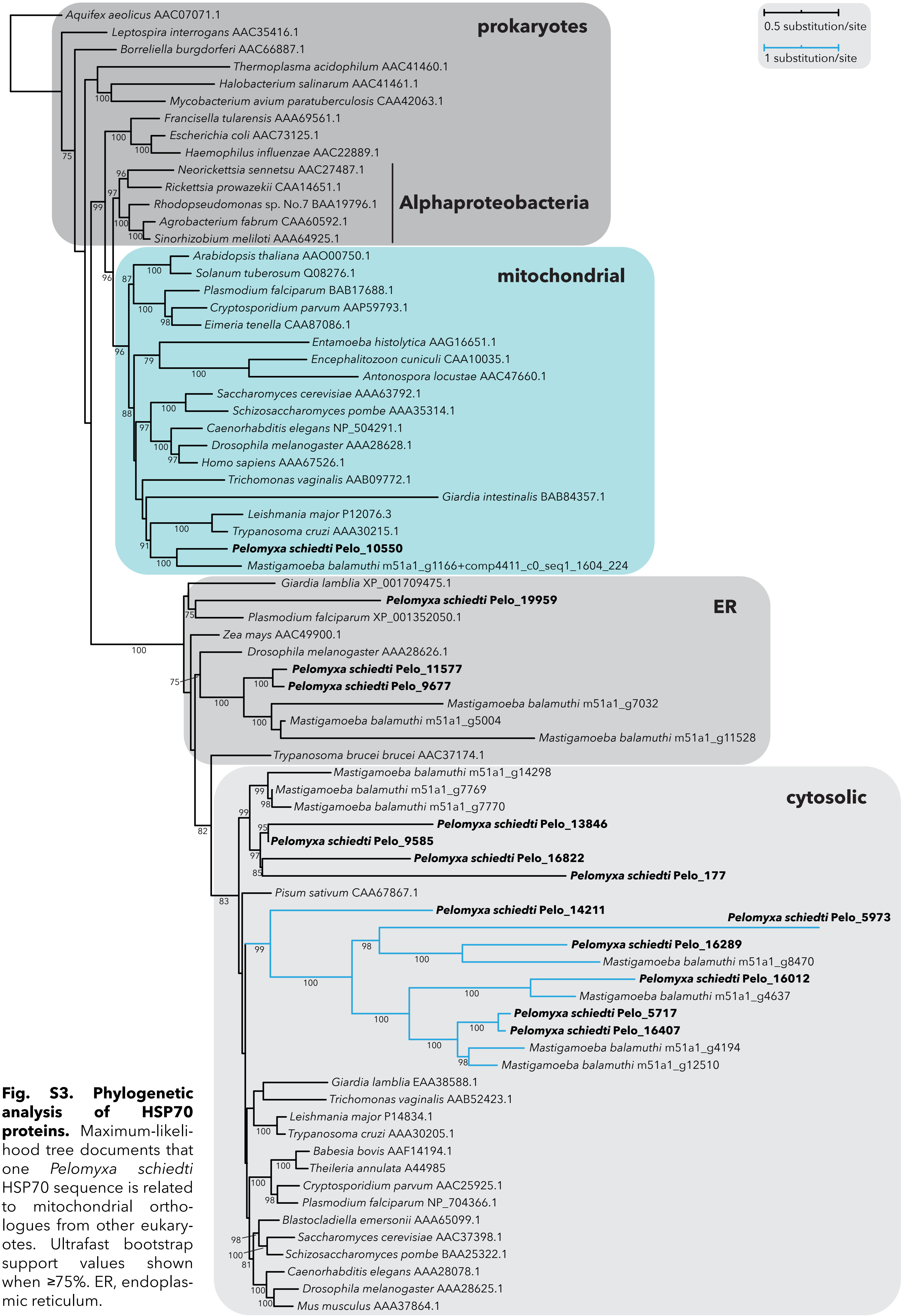

Fig. S3. Phylogenetic analysis of HSP70 proteins. Maximum-likelihood tree documents that one *Pelomyxa schiedti* HSP70 sequence is related to mitochondrial orthologues from other eukaryotes. Ultrafast bootstrap support values shown when ≥75%. ER, endoplasmic reticulum.

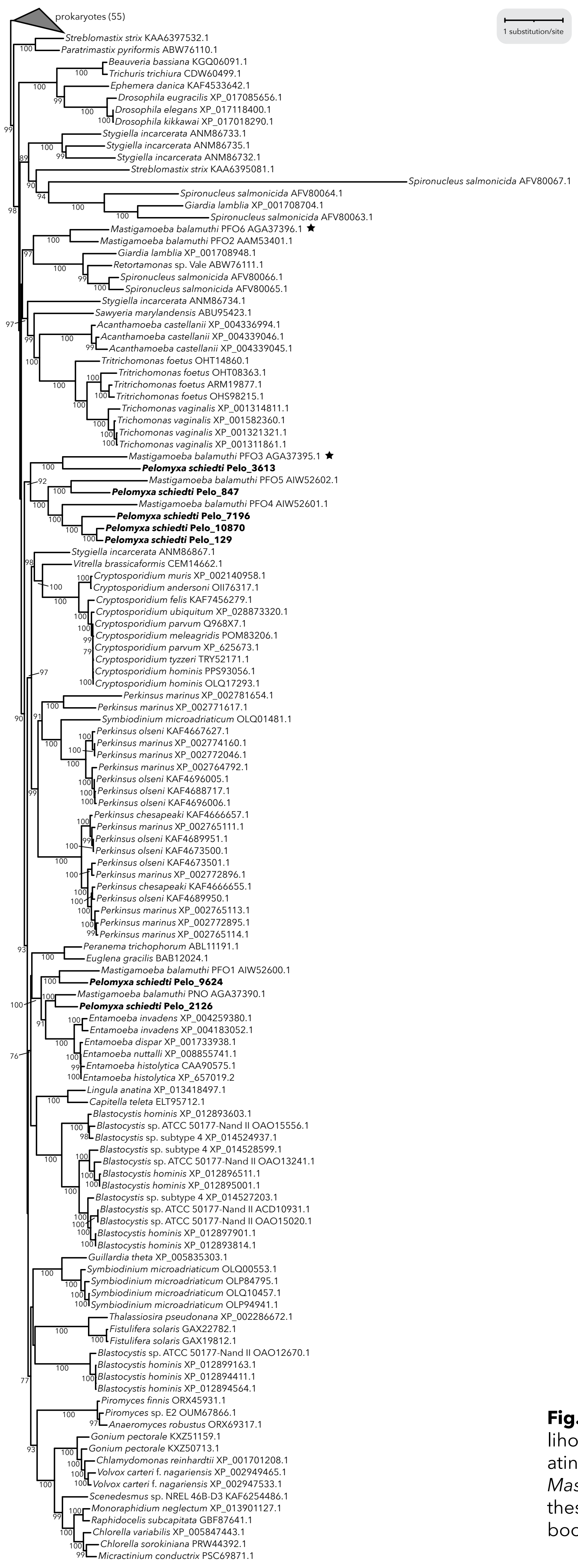

Fig. S4. Phylogenetic analysis of PFO enzymes. Maximum-likelihood phylogenetic tree identifies PFO version putatively operating in *Pelomyxa schiedti* MRO. Hydrogenosomal PFO copies of *Mastigamoeba balamuthi* marked with stars. Number in parentheses shows number of species in the collapsed clade. Ultrafast bootstrap support values shown when ≥75%.

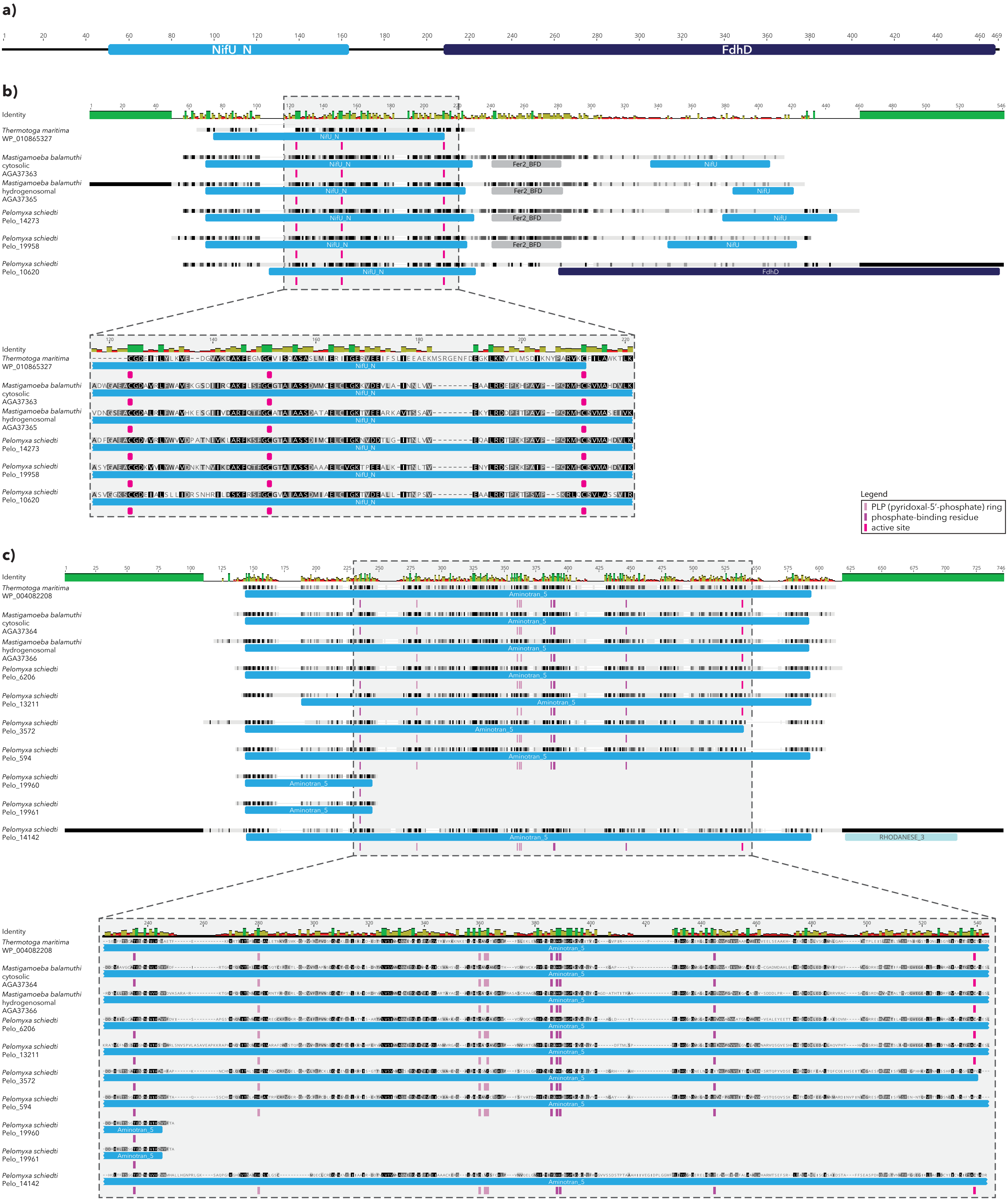

Fig. S5. Sequences of *Pelomyxa schiedti* components of NIF system. a) *P. schiedti* protein Pelo_10620, composed of NifU N-terminal domain fused to FdhD (formate dehydrogenase accessory sulfurtransferase) C-terminal domain, as determined by InterProScan. **b-c)** Sequence alignment of NifU (**b**) and NifS (**c**) proteins from *P. schiedti* and *Mastigamoeba balamuthi* in comparison with bacterial homologues from *Thermotoga maritima*. Amino acid residues necessary for function of NifU and NifS are labeled according to graphical legend.

a)

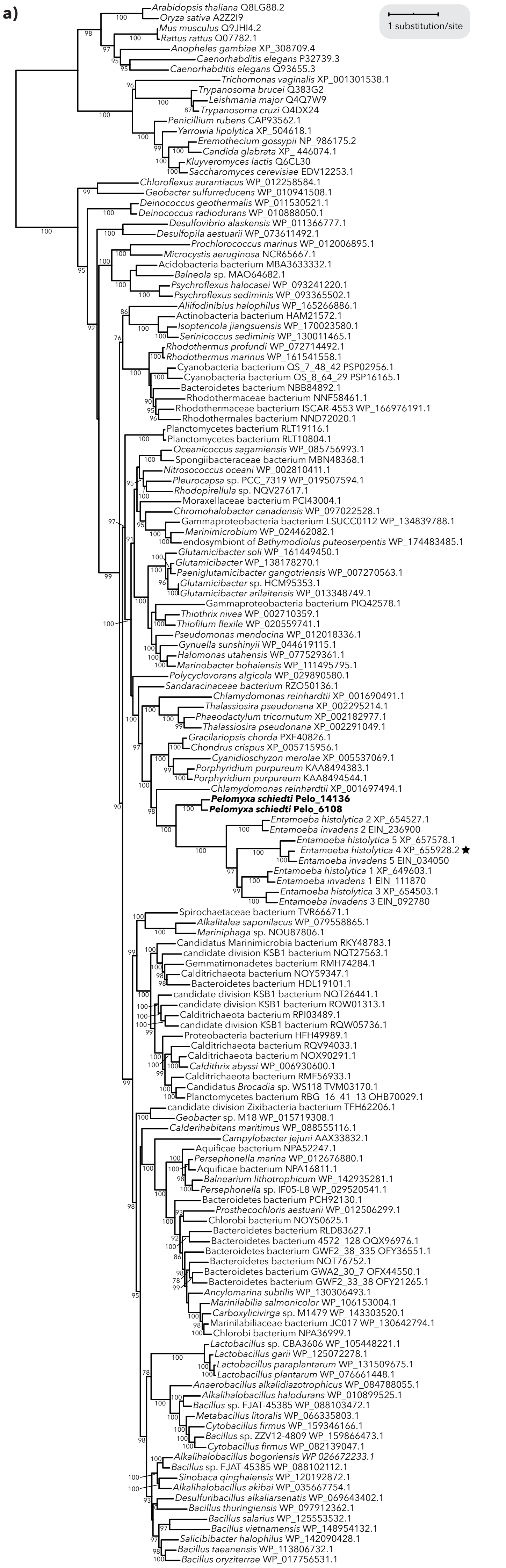

b)

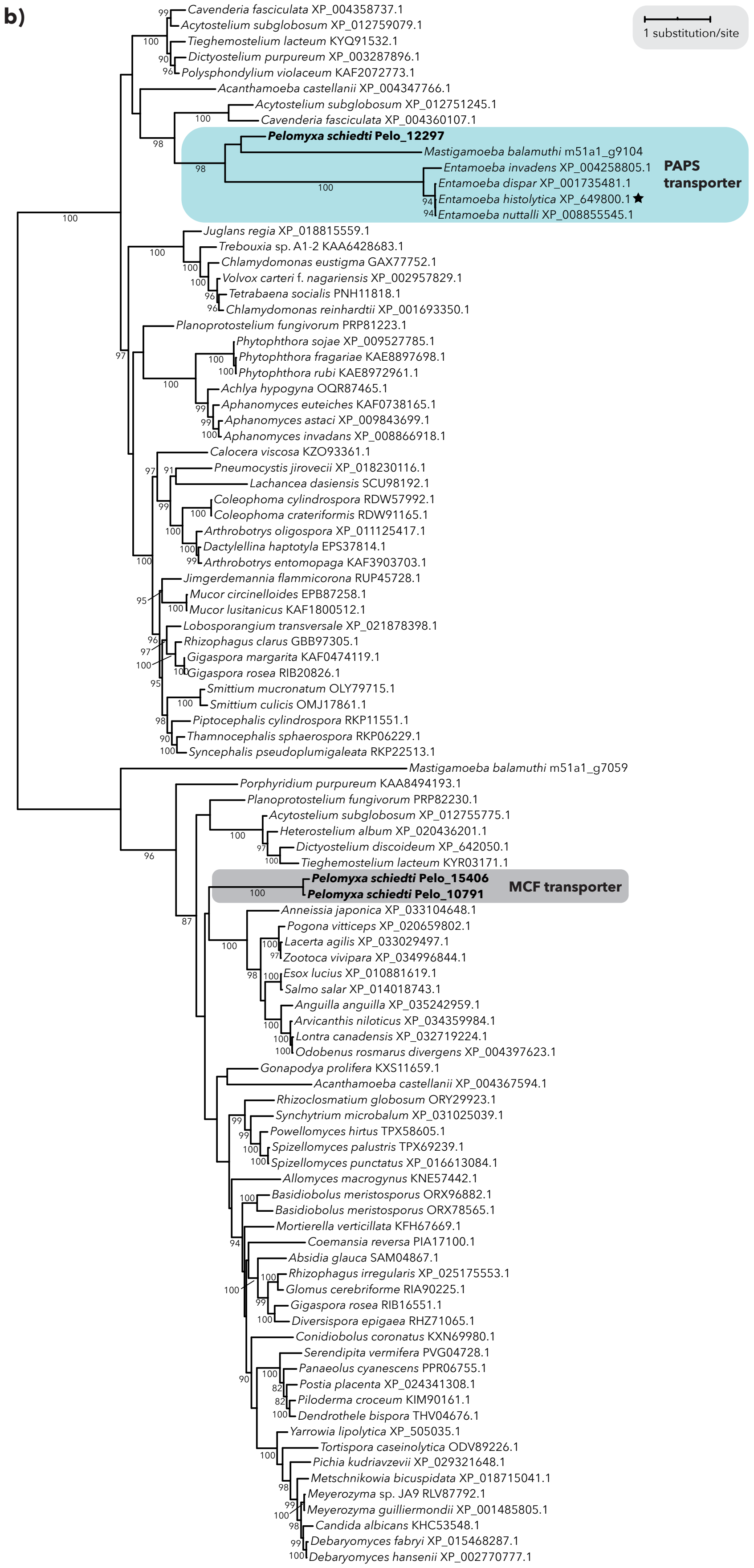

Fig. S6. Phylogenetic analysis of transporters involved in sulfate activation pathway. a) Phylogenetic analysis did not resolve which one of the sodium/sulfate symporters of *Pelomyxa schiedti* is related to *Entamoeba histolytica* mitosomal transporter. **b)** Maximum-likelihood phylogenetic tree confirms one *P. schiedti* transporter as PAPS (3′-phosphoadenosine 5′-phosphosulfate) transporter, while two others belong to broader family of mitochondrial carrier transporters. Experimentally proven mitosomal transporters of *E. histolytica* marked with stars. Ultrafast bootstrap support values shown when ≥75%.
